# Supplementary material for: Electron pairing and nematicity in LaAlO3/SrTiO3 nanostructures
Source: Nat Commun. 2023 Nov 23;14:7657. doi: 10.1038/s41467-023-43539-x (PMC10667274; doi:10.1038/s41467-023-43539-x)
Supplement: Supplementary file 1 — Supplementary information [file 41467_2023_43539_MOESM1_ESM.pdf]

Supplementary Information:

Electron Pairing and Nematicity in $LaAlO_3/SrTiO_3$ Nanostructures

Aditi Nethwewala[1,2], Hyungwoo Lee[3], Jianan Li[1,2], Megan Briggeman[1,2], Yun-Yi Pai [1,2], Kitae Eom[3], Chang-Beom Eom[3], Patrick Irvin[1,2], Jeremy Levy[1,2*]

[1]Department of Physics and Astronomy, University of Pittsburgh, Pittsburgh, PA 15260, USA.
[2]Pittsburgh Quantum Institute, Pittsburgh, PA, 15260 USA.
[3]Department of Materials Science and Engineering, University of Wisconsin-Madison, Madison, WI 53706, USA.

*Prof. Jeremy Levy, Department of Physics and Astronomy, University of Pittsburgh.
Email: jlevy@pitt.edu

This PDF file includes:

Supplementary Notes
Supplementary Fig. 1 to Supplementary Fig. 8
Supplementary References

Supplementary Notes

Finite Bias Spectroscopy and "Lever arm" Ratio

The conversion factor that relates changes in gate voltage to changes in chemical potential is known as the "lever arm" ratio [1]. It can be calculated by analyzing the nonlinear current-voltage relation of a device as a function of the applied gate voltage. The lever arm ratio, α is defined by $\delta\mu = \alpha\ \delta V_{sg}$ where δV_{sg} represents a change in the applied gate voltage and $\delta\mu$ denotes the resulting change in chemical potential. The horizontal red arrow in Supplementary Fig. 1 (b) marks the transition from one subband to another due to bias ΔV_{4T}. The energy gain induced by V_{4T} should be equal to the subband spacing marked by the vertical red arrow, $\alpha\Delta V_{sg}$ at zero bias, namely $e\Delta V_{4T}$ = $\alpha\Delta V_{sg}$. Then $\alpha = \ eV_{4T}/\Delta V_{sg}$ can be precisely calculated [2]. For Device A1 shown in Supplementary Fig. 1(b), $\alpha =$ $5.0\ \mu eV/mV$.

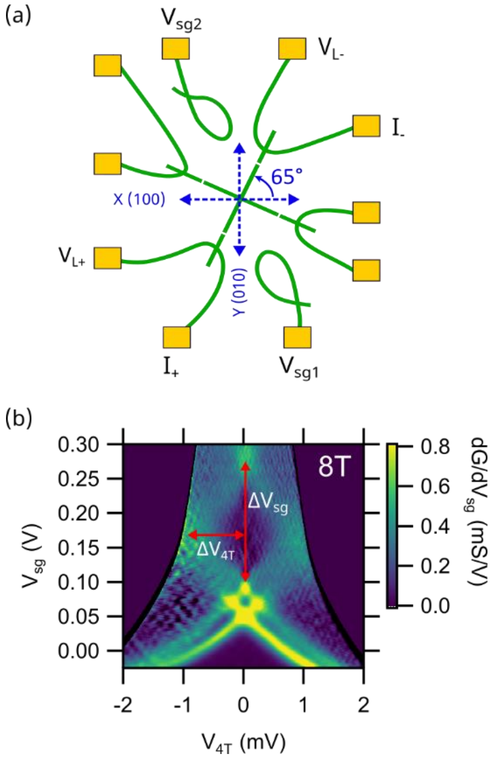

Supplementary Fig. 1: Finite Bias Spectroscopy on Device A1 oriented at $\varphi = 65°$ (a) Schematic showing the device geometry, (b) The transconductance map, shows the diamond feature characteristic of ballistic transport. The red arrows denote the parameters used to calculate the lever arm.

Longitudinal measurements across nanocross Device A1 oriented at $\varphi = 65°$ (full chemical potential range)

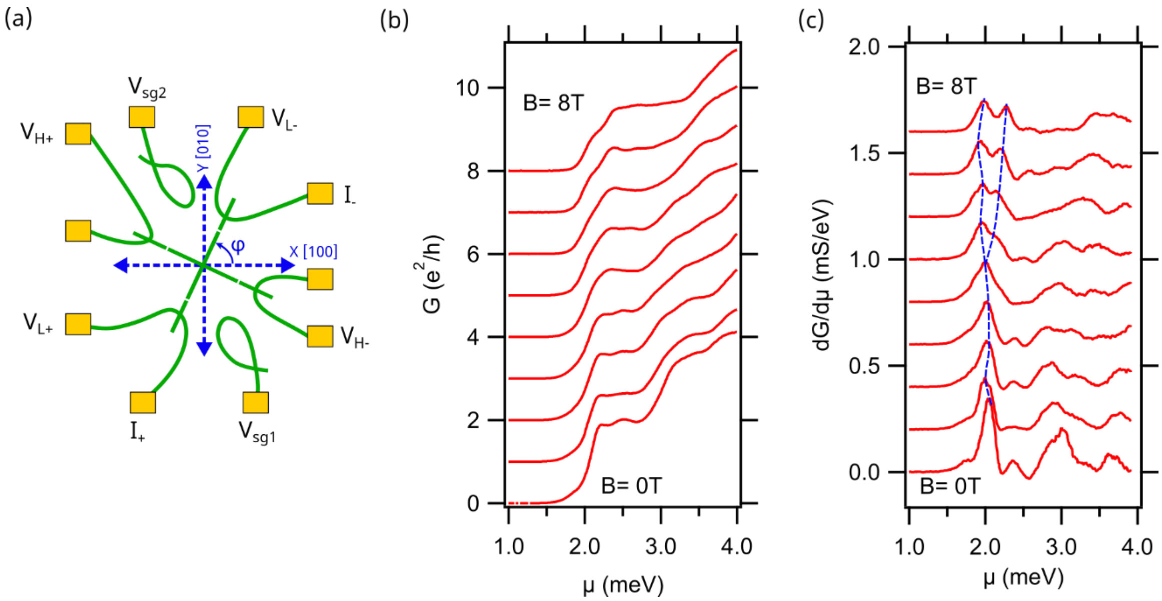

Supplementary Fig. 2: Nanocross device geometry and longitudinal measurements across nanocross Device A1 oriented at $\varphi = 65°$. (a) Schematic of longitudinal and Hall transport measurements across the nanocross. Angle φ denotes the relative orientation of the nanocross with respect to the crystallographic direction. Longitudinal voltage probes ($V_{L\pm}$) enable four-terminal conductance to be measured while transverse voltage probes ($V_{H\pm}$) enable Hall measurements. Both longitudinal and Hall measurements are acquired simultaneously as a function of gate voltage (V_{sg1} or V_{sg2}) and applied magnetic field, B, (b) Longitudinal conductance G versus chemical potential μ for magnetic fields ranging between $B = 0$ T and $B = 8$ T in steps of 1 T for Device A1 oriented at $\varphi = 65°$ with respect to [100] crystallographic direction. A conductance plateau near $G \approx 1.75\ e^2/h$ appears at all magnetic fields. For magnetic fields larger than $B = 4$ T, the transition to this plateau broadens significantly, and a second plateau is clearly visible at $G \approx 0.90 \pm 0.05\ e^2/h$ at $B = 8$ T. Curves are offset by $1\ e^2/h$ for clarity, (c) Transconductance dG/dμ versus μ for magnetic fields ranging between $B = 0$ T and $B = 8$ T in steps of 1 T and full range of chemical potential μ. dG/dμ versus μ reveals a transition between paired and unpaired state near $B = 4$ T as shown by the dashed blue lines. Curves are offset for clarity.

Variation of Hall resistance as a function of chemical potential for Device A1

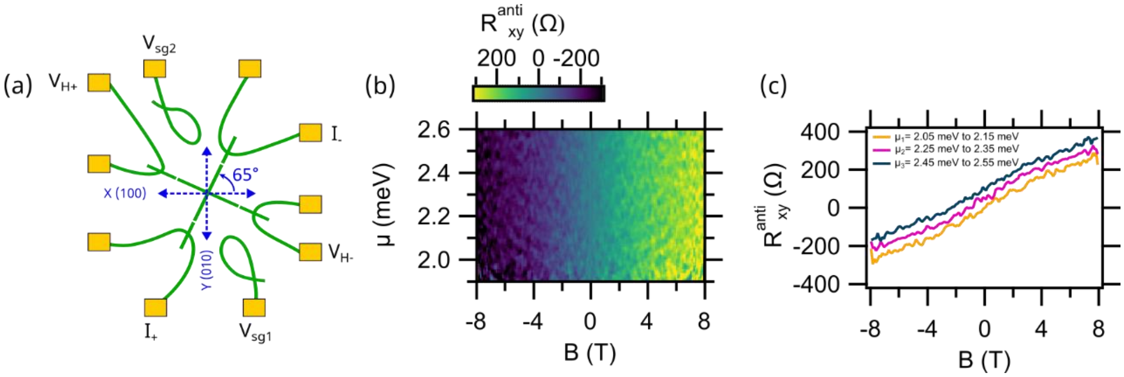

Supplementary Fig. 3:(a) Schematic showing the current and voltage leads for Hall measurement across the nanocross Device A1 oriented at $\varphi = 65°$, (b) Field anti-symmetrized Hall resistance, R_{xy}^{anti}, intensity map as a function of μ and B, (c) Line cuts of R_{xy}^{anti} as a function of B averaged between different ranges of chemical potential. The line cuts are shifted along the y-axis for clarity.

Hall measurements across nanocross devices A1, B1, C and D sculpted for $0° < \varphi < 90°$

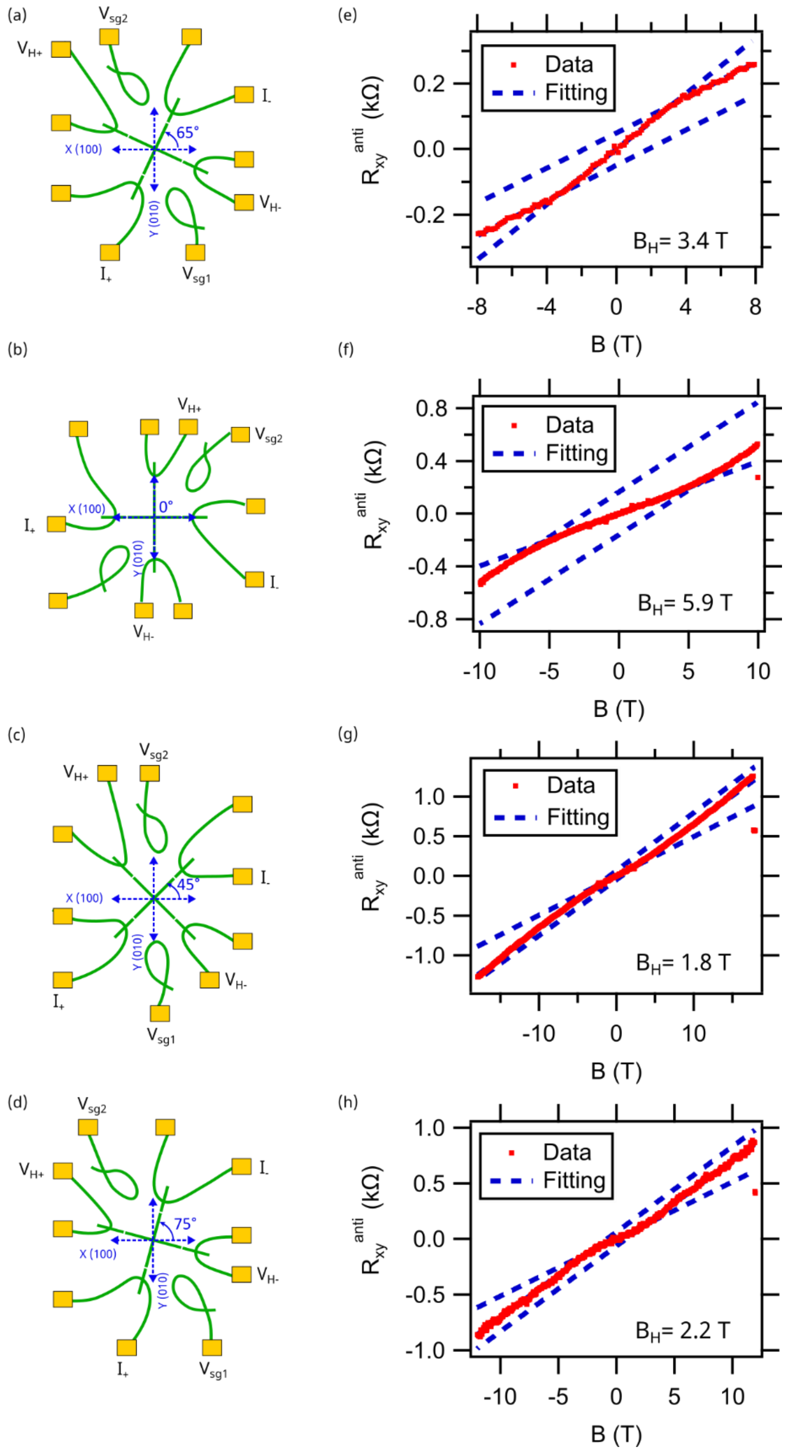

Supplementary Fig. 4: Hall measurements across nanocross devices A1, B1, C and D sculpted by varying φ between 0-degree and 90-degree with respect to the [100] crystallographic direction. (a-d) Schematic showing the current and voltage leads for Hall measurement across nanocross devices A1, B1, C and D oriented at $\varphi = 65°, 0°, 45°$ and $75°$ respectively, (e-h) Anti-symmetrized Hall resistance across nanocross devices A1, B1, C and D. Blue dashed lines show low-B and high-B asymptotes that cross at the Hall transition field B_H.

Reproducibility of Hall measurements for nanocross devices (A2, A3) and B2 created at the same location and orientation on the sample during different c-AFM lithography cycles.

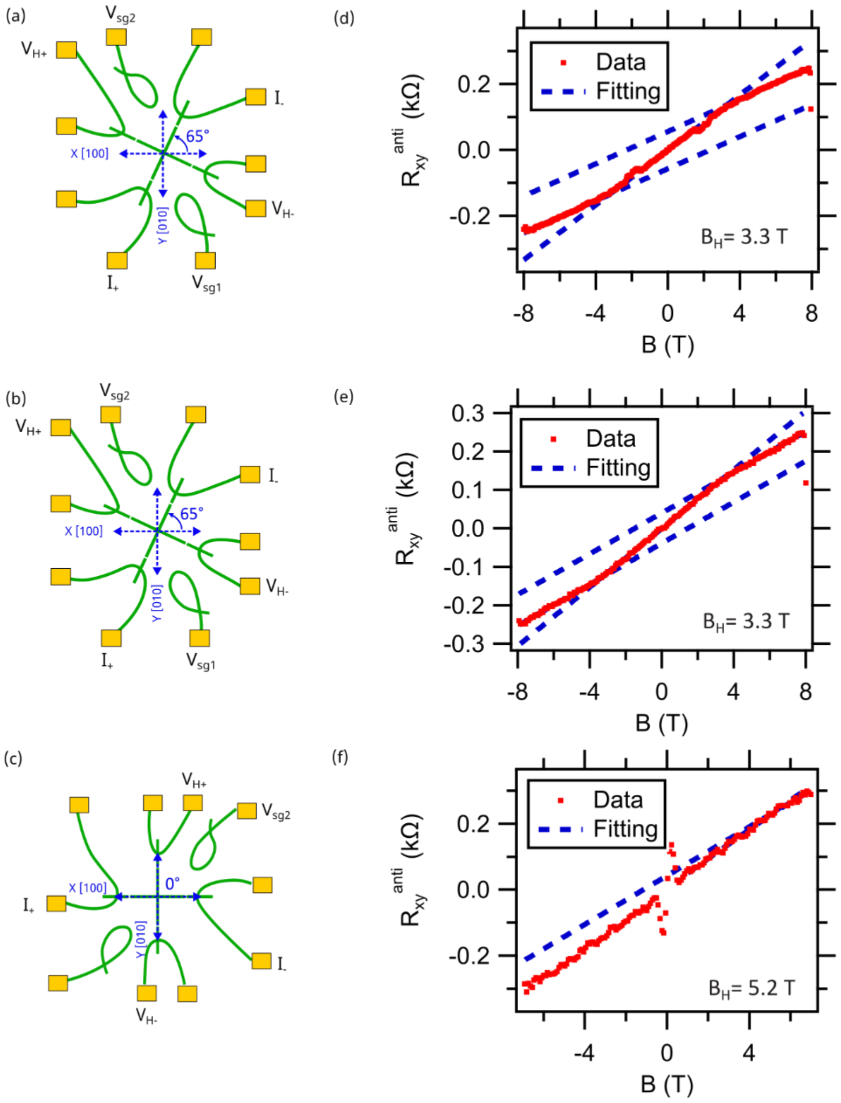

Supplementary Fig. 5: Reproducibility of Hall measurements across nanocross devices (A2, A3) and B2 written at the same location and orientation on the sample as devices A1 and B1 respectively during different c-AFM lithography cycles. (a-c) Schematic showing the current and voltage leads for Hall measurement across nanocross devices A2, A3, and B2 oriented at $\varphi = 65°$, 65°, and 0°, respectively, (d, e) Hall resistance across nanocross devices A2 and A3 oriented at $\varphi = 65°$, (f) Hall resistance across nanocross device B2 oriented at $\varphi = 0°$. Blue dashed lines show low-B and high-B asymptotes that cross at the Hall transition field B_H.

Conductance and transconductance measurements on Device E Sample 2 oriented at $\varphi = 45°$

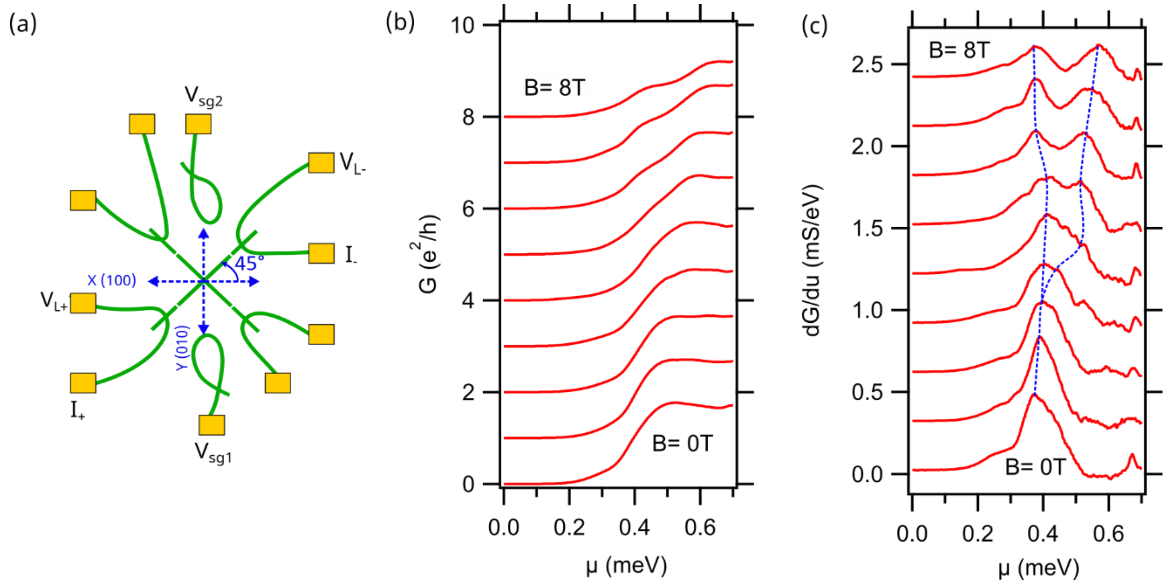

Supplementary Fig. 6: Nanocross device geometry and longitudinal measurements across nanocross Device E Sample 2 oriented at $\varphi = 45°$. (a) Schematic of longitudinal and Hall transport measurements across the nanocross. Angle φ denotes the relative orientation of the nanocross with respect to the crystallographic direction. Longitudinal voltage probes ($V_{L\pm}$) enable four-terminal conductance to be measured while transverse voltage probes ($V_{H\pm}$) enable Hall measurements. Both longitudinal and Hall measurements are acquired simultaneously as a function of gate voltage (V_{sg1} or V_{sg2}) and applied magnetic field, B, (b) Longitudinal conductance G versus chemical potential μ for magnetic fields ranging between $B = 0$ T and $B = 8$ T in steps of 1 T on device E oriented at $\varphi = 45°$ on sample 2. A conductance plateau near $G \approx 1.70\, e^2/h$ appears at all magnetic fields. For magnetic fields larger than $B = 2$ T, the transition to this plateau broadens significantly and a second plateau is clearly visible at $G \approx 0.90 \pm 0.05\, e^2/h$ at $B = 6$ T. Curves are offset by $1\, e^2/h$ for clarity. (c) Transconductance $dG/d\mu$ versus μ for magnetic fields ranging between $B = 0$ T and $B = 8$ T in steps of 1 T. $dG/d\mu$ versus μ reveals a transition between paired and unpaired state near $B = 2$ T as shown by the dashed blue lines. Curves are offset for clarity.

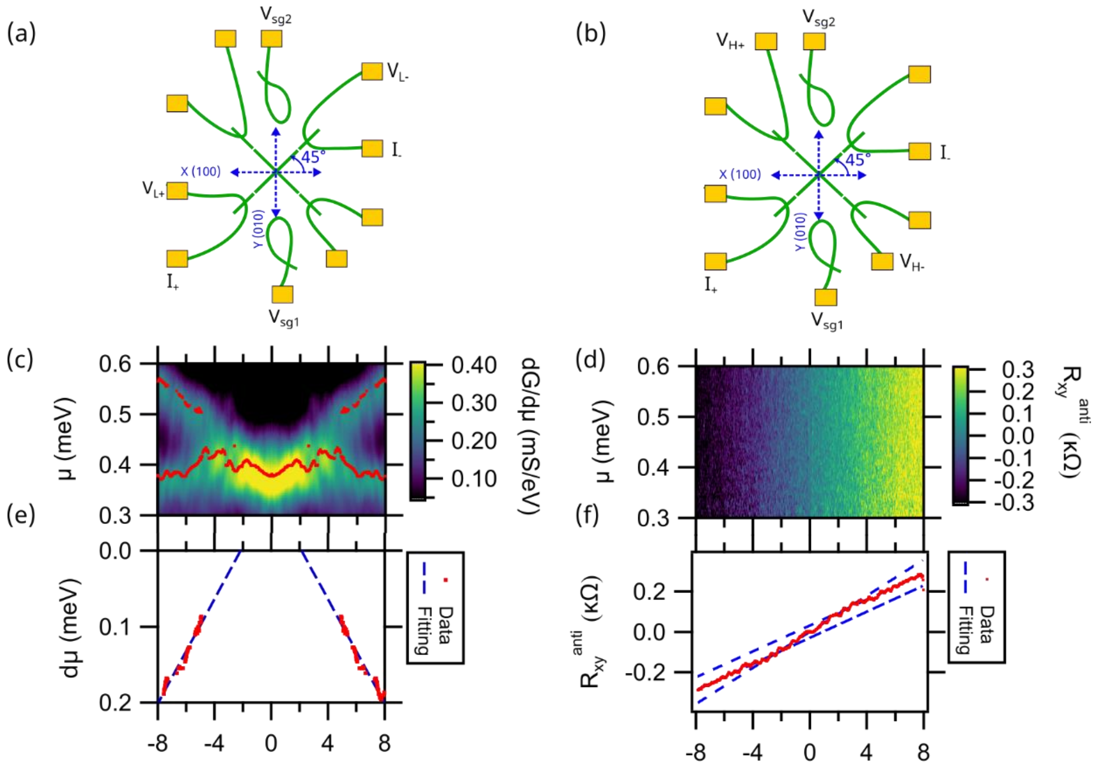

Supplementary Fig. 7: Comparison of transconductance $dG/d\mu$ and Hall measurements on Device E sample 2 oriented at $\varphi = 45°$. (a, b) Schematic showing the current and voltage lead configurations for longitudinal and Hall measurement across nanocross device E. (c) Intensity plot of transconductance $dG/d\mu$ versus chemical potential μ and magnetic field B. Fits to peak of transconductance versus magnetic field are overlaid. The splitting in the transconductance from a single peak to two peaks is characteristic of the electron pairing transition. (d) Intensity plot of Hall resistance R_{xy}^{anti} versus μ and B. (e) Plot of energy difference between transconductance peaks versus magnetic field. Blue dashed line extrapolates to a value of $B_P = 2.2 \pm 0.4$ T. (f) Average Hall resistance over the range $\mu = 0.51$ meV to 0.52 meV reveals nonlinear behavior with asymptotes that cross at $B_H = 2.4 \pm 0.6$ T.

Angle dependence of Hall slope R_H and transition field B_H in LAO/STO.

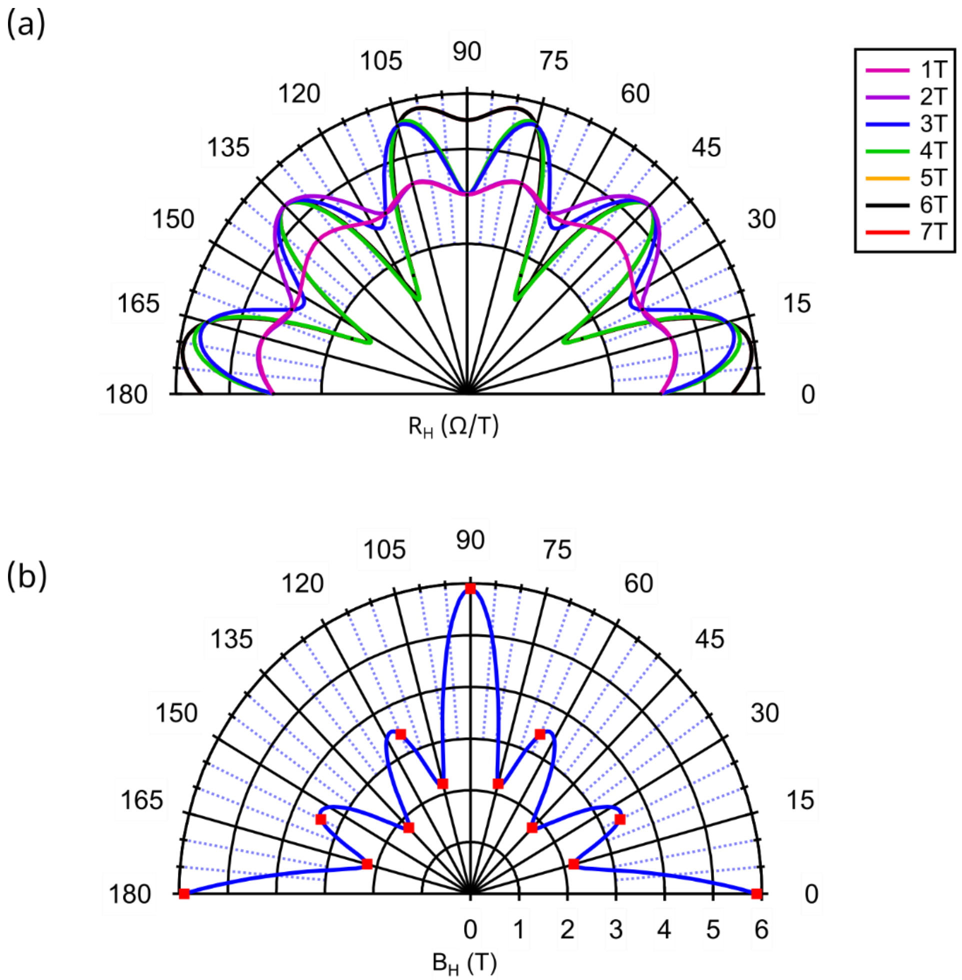

Supplementary Fig. 8: Angle dependence of Hall slope R_H and Hall transition field B_H in LAO/STO at increasing magnetic field strengths. Angle φ denotes the relative orientation of the nanocross with respect to the [100] crystallographic direction. (a) Spline fit showing variation in R_H with increasing magnetic field strength, $1\ T \leq B \leq 7\ T$ for $0 \leq \varphi \leq 180°$ (b) Spline fit showing variation in critical magnetic field, B_H for $0 \leq \varphi \leq 180°$. The graphs consider two axes of symmetry, rotational symmetry by $90°$ and mirror symmetry along $45°$.

References

[1] R.J. Warburton, B.T. Miller, C.S. Dürr, C. Bödefeld, K. Karrai, J.P. Kotthaus, G. Medeiros-Ribeiro, P.M. Petroff, S. Huant, Coulomb interactions in small charge-tunable quantum dots: A simple model, Physical Review B, 58 (1998) 16221-16231.
[2] A. Annadi, G. Cheng, H. Lee, J.-W. Lee, S. Lu, A. Tylan-Tyler, M. Briggeman, M. Tomczyk, M. Huang, D. Pekker, C.-B. Eom, P. Irvin, J. Levy, Quantized Ballistic Transport of Electrons and Electron Pairs in $LaAlO_3/SrTiO_3$ Nanowires, Nano Lett, 18 (2018) 4473-4481.
